# Factors affecting the accuracy of genomic selection for growth and wood quality traits in an advanced-breeding population of black spruce (*Picea mariana*)

**DOI:** 10.1186/s12864-017-3715-5

**Published:** 2017-04-28

**Authors:** Patrick R.N. Lenz, Jean Beaulieu, Shawn D. Mansfield, Sébastien Clément, Mireille Desponts, Jean Bousquet

**Affiliations:** 1 Canadian Wood Fibre Centre, Canadian Forest Service, Natural Resources Canada, Government of Canada, 1055 du PEPS, P.O. Box 10380, Québec, Québec G1V 4C7 Canada; 2 Canada Research Chair in Forest Genomics, Institute of Systems and Integrative Biology and Centre for Forest Research, Université Laval, 1030, Avenue de la Médecine, Québec, Québec G1V 0A6 Canada; 3 Department of Wood Science, Forest Sciences Centre, University of British Columbia, Vancouver, British Columbia V6T 1Z4 Canada; 4 Ministère des Forêts, de la Faune et des Parcs, Gouvernement du Québec, Direction de la recherche forestière, 2700 rue Einstein, Québec, Québec G1P 3W8 Canada

**Keywords:** Genomic selection, Black spruce, Wood properties, Tree improvement and breeding, Genomic-estimated breeding values, Gene SNPs

## Abstract

**Background:**

Genomic selection (GS) uses information from genomic signatures consisting of thousands of genetic markers to predict complex traits. As such, GS represents a promising approach to accelerate tree breeding, which is especially relevant for the genetic improvement of boreal conifers characterized by long breeding cycles. In the present study, we tested GS in an advanced-breeding population of the boreal black spruce (*Picea mariana* [Mill.] BSP) for growth and wood quality traits, and concurrently examined factors affecting GS model accuracy.

**Results:**

The study relied on 734 25-year-old trees belonging to 34 full-sib families derived from 27 parents and that were established on two contrasting sites. Genomic profiles were obtained from 4993 Single Nucleotide Polymorphisms (SNPs) representative of as many gene loci distributed among the 12 linkage groups common to spruce. GS models were obtained for four growth and wood traits. Validation using independent sets of trees showed that GS model accuracy was high, related to trait heritability and equivalent to that of conventional pedigree-based models. In forward selection, gains per unit of time were three times higher with the GS approach than with conventional selection. In addition, models were also accurate across sites, indicating little genotype-by-environment interaction in the area investigated. Using information from half-sibs instead of full-sibs led to a significant reduction in model accuracy, indicating that the inclusion of relatedness in the model contributed to its higher accuracies. About 500 to 1000 markers were sufficient to obtain GS model accuracy almost equivalent to that obtained with all markers, whether they were well spread across the genome or from a single linkage group, further confirming the implication of relatedness and potential long-range linkage disequilibrium (LD) in the high accuracy estimates obtained. Only slightly higher model accuracy was obtained when using marker subsets that were identified to carry large effects, indicating a minor role for short-range LD in this population.

**Conclusions:**

This study supports the integration of GS models in advanced-generation tree breeding programs, given that high genomic prediction accuracy was obtained with a relatively small number of markers due to high relatedness and family structure in the population. In boreal spruce breeding programs and similar ones with long breeding cycles, much larger gain per unit of time can be obtained from genomic selection at an early age than by the conventional approach. GS thus appears highly profitable, especially in the context of forward selection in species which are amenable to mass vegetative propagation of selected stock, such as spruces.

## Background

Genomics of forest trees is rapidly gaining momentum as it promises to unravel the genetic control of adaptive and economically important traits, in part to satisfy the increasing demand for high quality wood fibre worldwide but also, to cope with the increasing challenges imposed by changing climates and environments [1, 2]. Uses of genomic information for breeding are diverse and may rely on reconstruction of the pedigree, verification of co-ancestry in breeding populations for genetic diversity management purposes, or on the correlation of marker information with phenotypes for selection [3]. For example, marker-assisted selection (MAS) was among the first approaches suggested to accelerate tree improvement [4]. In conifers, with the use of current statistical methods and correction for multiple testing, a limited number of markers or genes have been reported to be genetically linked to economically important traits, such as wood quality and/or tree growth [5–7]. Individual Single Nucleotide Polymorphism (SNP) markers are largely constrained to explaining only a minor proportion of the variation, as they rarely explain more than 5% of quantitative trait variation [5–9]. Therefore, for most quantitative traits, the association approach does not appear useful enough in breeding selection where accurate predictions of genetic values are necessary. When using large progeny sets, the proportion of trait variance explained by individual quantitative trait loci (QTLs) has only been slightly higher, which is consistent with the multigenic control of these traits [9–11]. Additionally, the weak linkage disequilibrium (LD) between markers and QTLs across different genetic backgrounds, as well as the narrow range captured in typical QTL studies limits their use to within family selection.

To overcome the limitations of MAS, genomic selection (GS) uses dense genomic marker information (called genomic signatures or profiles) of individuals, as well as their parents when available, to predict their breeding value [12]. Contrary to MAS, GS models simultaneously estimate the effects of all available markers in a training population. Predictions or genomic-estimated breeding values (GEBVs) are then made for progeny of the same or future generations [12]. One of the basic assumptions is that the markers are scattered throughout the genome so that at least some of them are in direct linkage with causal loci [13].

Most economically important traits are of quantitative nature and controlled by many genes [2]. Consequently, it is crucial that an adequate number of markers are used to attain sufficient coverage of the genome, and that as many loci as possible are in LD with QTLs [14]. The combination of the rapidly decreasing costs of high-throughput genotyping as well as the significant advances in sequencing and subsequent computation has facilitated GS-based selection approaches to be considered in organisms even with very large genomes, such as trees and conifers, in particular.

Initially employed in dairy cattle breeding [12, 14, 15], GS has also been applied to other animals such as mice [16], and in plant and crop breeding [17–19]. In trees, GS has been tested in both angiosperms such as eucalypts [20], and gymnosperms such as loblolly pine (*Pinus taeda* L.) [21, 22], maritime pine (*Pinus pinaster* Aiton), [23, 24] and white spruce and its hybrids (*Picea glauca* (Moench) Voss) [25–28]. However, studies on boreal conifer species are still relatively rare, which is most likely due to the fact that substantive genomic resources were only recently made available for several species (reviewed by De La Torre et al. [29]).

Generally, tree improvement of boreal conifer species is characterized by breeding cycles of 30 years and longer, which includes production of crosses, field evaluation of progeny and performing selections, and the propagation of selected superior material through sexual or vegetative means [30]. Early selection methods that facilitate accurate prediction of mature phenotypes at a younger stage are therefore vital to shorten breeding cycles and ultimately improve the cost-efficiency of such breeding programs. Traditionally, these methods have relied on indirect methods of phenotypic selection, which may be less effective, especially when genetic correlations between the juvenile and mature traits are low [31]. Performing selection directly on genomic marker information would, in theory, avoid the loss of prediction accuracy due to imperfect correlation between different life stages, and would mostly be dependent on the heritability of the target trait at the mature stage [32].

Genomic selection promises to significantly reduce the time commitment for completing a breeding cycle and thus to increase genetic gain per time unit by avoiding the field testing stages, that often represent the largest time commitment [3, 32]. Technically, the estimates of breeding values could be obtained at the seedling stage or even from seed or somatic tissue [1]. Hence, the time required to select elite genotypes of northern conifers, which is largely impacted by their slow inherent growth could be overcome, and mature phenotypes would only be needed for model construction and validation [26].

Previous GS studies in forest trees have reported moderate to high accuracy of selection models with correlations of between 0.6 and 0.8 when comparing GEBVs with conventional EBVs, when full-sib families were considered [20, 25, 33]. This suggests that under certain conditions of high relatedness where long-range linkage disequilibrium (LD) is likely more a potent factor of accuracy than short-range LD, the GS approach can easily compete with the conventional pedigree-based breeding approach or even outperform it due to the reduction in time needed to complete a breeding cycle [25, 32, 33]. In contrast, lower accuracy values (0.3 and 0.5) have been observed when half-sib families were considered [26, 28], and GS models developed in unrelated trees had very low power to make prediction [25, 26]. The marginal to low performance of these latter two cases of GS is linked to the larger effective population size and low LD, which is common to natural populations of undomesticated trees [34–36]. Large population sizes indeed results in more recombination and a greater diversity among available gametes within populations, from high outcrossing rates in wind-pollinated species such as black spruce [37]. Moreover, the genetic drift common to small populations with fewer parents generating non-random associations among alleles at different loci, i.e., gametic disequilibrium or LD [38], is not a significant factor in such conditions.

Black spruce (*Picea mariana* [Mill.] B.S.P.) is one of the most abundantly distributed trees throughout the transcontinental boreal forest of Canada and Alaska. It is also one of the most reforested species in Canada, with 65 million seedlings being planted per year in the province of Québec alone, and has been the subject of advanced-generation breeding programs [30].

The objectives of this study were to (1) estimate accuracies and gains per unit of time derived from GS for wood quality and growth traits in an advanced-breeding population of the black spruce; (2) assess variation in accuracy from building site-specific or multiple-site models; (3) explore the roles of relatedness, short- and long-range linkage disequilibrium in genomic prediction accuracy by simulating different family structures and testing different subsets of markers; and (4) investigate the effect of sample sizes on model accuracy.

To meet these objectives, we used trait data obtained from 25-year-old black spruces grown in a genetic test replicated on two environmentally contrasted sites in Québec Canada. The genetic test consisted of full-sib families generated using a partial diallel mating design. Trees were genotyped for 4993 SNP markers representative of as many distinct gene loci, and originating from a black spruce high-confidence SNP catalogue previously assembled and validated [39].

## Methods

### Sampling of plant material

Phenotypic and genetic data were obtained for 734 25-year-old progeny trees belonging to 34 controlled-pollinated families derived from a partial diallel mating design that consisted of 27 parents originating from 9 provenances from the Canadian provinces of Ontario, New Brunswick and Manitoba, and one from Maine in the northeastern United States. The parental trees had previously been identified for their superior growth and stem form in a range-wide provenance trial established on 4 sites in Québec [40]. Progeny were raised as cuttings in the nursery for three years and then established in 1991 by the Ministère des Forêts, de la Faune et des Parcs du Québec (MFFPQ) on two forest sites using a randomized complete block design, with 4-tree row plots and a 2 m by 2 m spacing. The test sites are located in two contrasting environments in the eastern part of the province of Québec: 1) the Matapedia arboretum (latitude: 48° 32’N, longitude: 67° 25’W, elevation 216 m, 333 trees sampled), which is characterized by a warmer climate typical of the balsam fir – yellow birch forest, and 2) the Robidoux site (latitude: 48° 18’N, longitude: 65° 31’W, elevation 275 m, 401 trees sampled), which is characterized by a colder climate typical of the balsam fir – white birch forest domain and under the influence of the Chic-Choc mountains (1270 m) on the Gaspésie peninsula. Needle tissues for DNA extraction were sampled in the upper third of the crown, immediately placed on ice and stored at −10 °C until further processing. DNA extraction protocols used for collected samples are described in detail in Pavy et al. [41].

### Phenotypic trait determination

Tree height and diameter at breast height (DBH, 1.3 m above ground) were recorded in 2013 at the age of 25 years, and a wood increment core was extracted from the south facing side of each tree. Cores were stored in a freezer, conditioned to 7% moisture and cut to 1.68 mm thickness prior to X-ray densitometry analyses (Quintek Measurement Systems, TN). Wood density was calculated as a ring area weighted mean from recorded pith to bark wood density profiles. Microfibril angle (MFA) was estimated using X-Ray diffraction, on a Bruker D8 Discovery unit equipped with an area array detector, as per Ukrainetz et al. [42].

### Genotyping

In recent years, significant genomic resources have been developed for white spruce and Norway spruce (*Picea abies* (L.) Karst.), including draft genome sequences [43–46], genetic and QTL maps [11, 47, 48], gene catalog and expression chips [49], large SNP registries and high throughput genotyping chips [40, 50], but comparatively few genomic resources exist for black spruce. Before investigating GS in black spruce, exome capture and sequencing were used together with an in-house bioinformatic pipeline to produce a registry of 97,000 high-confidence SNPs pertaining to around 15,000 gene sequence clusters [39]. In addition, we compiled successfully genotyped gene SNPs from previous black spruce genomic studies [9, 41, 51–53]. Altogether, this information was used to develop an Infinium iSelect SNP genotyping array (Illumina, San Diego, CA) containing 5,300 SNPs representing as many distinct black spruce gene contigs [39]. Based on control DNA replicates, the chip genotyping reproducibility rate obtained was 99.9%. For genomic selection modelling and analyses, genotyping information from a total of 4,993 SNPs representing as many distinct gene loci spread among the 12 spruce linkage groups was retained for each of the 734 progeny trees, resulting in a total of >3.6 million SNP calls. Given the estimated 1,850 centimorgans (cM) of the black spruce genome [51, 54], this corresponds to a marker density of approximately 2.7 per cM. For all 4,993 SNPs retained for GS analyses, the following in-house quality criteria were met: biallelic SNPs matching with both parental genotypes, a *GenTrain* (Illumina, San Diego, California) quality score ≥0.25, a call rate ≥85%, a fixation index |*F*_IS_| ≤ 0.50, and a minor allele frequency (MAF) ≥0.0055. Furthermore, SNPs with minor frequency alleles that were present in less than 10 individuals were not considered. Of the retained markers, only 2% had a minimum allele frequency (MAF) < 5%, indicating a small number of rare allele markers.

### Estimating "true" breeding values

For each trait, an individual tree (so called “animal”) model was fit using the GS3 software [55] in order to estimate reference or “true” breeding values:

1 $$ y= X\beta + S p+ T u $$

where, *β* is a vector of fixed effects, including an overall mean and the fixed site effect, *p* is the permanent random block effect, *u* is the vector of random additive polygenic effects following a distribution ~ *N*(0, *A*σ^2^_u_) and e is the error term with ~ *N*(0, *I*σ^2^_e_). *X*, *T* and *S* are incidence matrices, and *A* is the numerator of the relationship matrix describing the additive relationship among individuals and *I* is the identity matrix. All the trees sampled were used to estimate these reference breeding values.

### Basic genomic selection models with all information

In the first set of analyses, phenotypes from both sites were combined and GS models were fit using a simple Bayesian framework in the GS3 software [55, 56], considering all SNPs in the models. Phenotypes were standardized by block-within-site effects and site standard deviation in order to account for differences between sites. The genomic-estimated breeding value (GEBV) of each tree was estimated for all phenotypic traits using SNP marker information whose effect was estimated with the linear mixed model:

2 $$ y= X\beta + Z\alpha + e $$

where, *y* is the vector of standardized phenotypes, *β* is a vector of fixed effects including an overall mean, *α* is the random marker effect, *e* the random error, and *X* and *Z* are incidence matrices. The latter was built from the number of alleles observed for each individual and SNP, and coded as 0, 1 and 2 representing the number of copies of the minor allele. Approximately 1.8% of the total number of genotypes was missing and imputed as the mean of the respective marker rounded to the next genotype value. Given that only biallelic markers were retained for the analyses, values of +0.5*a*_*j*_ and −0.5*a*_*j*_ were arbitrarily assigned to alleles 1 and 2 respectively, which follows conventional parametrization where the difference between the two homozygotes equals two *a*_*j*_ [55]. Marker effects were assumed to follow a normal distribution ~ *N*(0, *I*σ^2^_a_), where *I* is the identity matrix. The model hence assumes common variance and all marker coefficients are minimized to the same extent, which is commonly called ridge regression. The approach was deemed appropriate for similar traits evaluated in white spruce and assumingly controlled by many genes of small effect [25], and was hence retained for all analyses in this study. GEBVs were estimated as

3 $$ {\widehat{\mathrm{g}}}_i={\displaystyle {\sum}_{j=1}^n Z{\hbox{'}}_{i j}{\hat{\mathrm{a}}}_j} $$

where, *Z’*_*ij*_ is the indicator co-variate (−1, 0 or 1) for the i^th^ tree at the j^th^ locus and *â*_*j*_ is the estimated effect at the j^th^ locus.

The GS3 software uses the Gauss-Seidel algorithm with residual update for best linear unbiased prediction (BLUP), and for best linear unbiased estimation of random and fixed effects respectively [55]. The algorithm is extended by Gibbs sampling for estimation of variance components. The Gibbs sampler was run for 100,000 iterations with a burn-in of 20,000 iterations. Every thousand iterations, a sample was retained and convergence of the posterior distribution was verified using trace plots. Flat priors were found to give the most stable results of convergence for the various models and subsamples tested.

### Model validation and estimation of accuracy

Tenfold cross-validation was performed for pedigree-based and marker-based models in order to obtain predicted breeding values for both approaches. For each round of cross-validation, 10% of the trees were randomly drawn from each family and set aside for validation, the remainder being used for model training. Each individual was thus included in one validation set. Model quality was evaluated by the accuracy, r(GEBV, EBV), which is defined here as the correlation between the cross-validated genomic-predicted breeding values and the “true” or reference breeding values previously calculated using pedigree information and all available sampled trees. The predictive ability, r(y, ŷ), was similarly evaluated as the correlation between predicted and actual phenotypes.

### Testing site specificity

Different GS scenarios were also tested to investigate the influence of site on model quality: the basic model contained all 734 trees from the two sites combined (see above); another set of models were trained and validated with only data from a single site, and the last models were trained with data from one site and validated with data from the second site.

### Testing the effect of relatedness on accuracy estimates

In order to address questions related to the influence of relatedness and long-range linkage disequilibrium (LD) on genomic selection accuracy, we investigated the effect of family structure. Training and validation sets were genetically related in the basic models, *i.e.* different trees, but originating from the same full-sib families. For comparison purposes, a genomic selection scenario with half-sib families was also tested where the training and validation sets shared female progenitors, but form different families, hence implicating different males, or vice versa. Estimates of model accuracy were also obtained by using completely unrelated trees from families and provenances excluded from model training.

### Testing subsets of markers

To investigate the effect of LD, and that of various subsets of markers on GS model accuracy, different subsets were delineated and used to build models. Hence, genomic selection models were constructed with subsets of 1,000, 500 and 250 markers randomly drawn from the 4,993 SNPs available, as well as with a set of 250 markers having the largest absolute effects and a set containing the 4,743 remaining markers. Accuracy and predictive ability of these reduced models were then compared with those of the basic model. GS models were also constructed separately with markers identified for each of the 12 black spruce linkage groups, in order to investigate the contribution of various genomic regions to the genetic control of the different traits and the influence of LD on GS model accuracy. Given the very high synteny and collinearity between the white spruce and black spruce genomes [51], we used the more complete linkage mapping information of white spruce gene homologs to approximate the genomic positions of black spruce genes carrying the SNPs considered herein. Of the 4,993 black spruce gene contigs of the present study (with gene nomenclature according to the white spruce gene catalogue of Rigault et al. [49] as described in Pavy et al. [39]), 2,928 genes had homologs mapped on the most recent white spruce reference genetic map containing nearly 9,000 genes [48]. These homologs were well distributed over the 12 linkage groups with, on average, 244 (+/- 15) gene homologs represented per chromosome.

### Testing the size of training data set

Random subsets of individuals of various sizes were used to examine the minimum number of trees needed to build GS models without significantly loosing accuracy. Starting with the complete sample set of 4,993 SNPs and 734 trees, about one third of the trees was iteratively removed, creating subsets of 490, 330, 224, 147 and 106 trees that were subsequently analysed with all the 4,993 SNPs.

### Genetic gain estimations

Estimates of genetic gain from conventional selection or from GS were obtained by using predicted breeding values for each trait and by considering a selection intensity of 5%. Gains per year were obtained by assuming a spruce breeding cycle of a minimum of 28 years for conventional selection, and a shortened cycle of 9 years for GS, with 4 years for crosses and production of seedlots that are full-siblings to the training population, followed by 1 year for selection of individuals using markers and genomic selection models, and 4 years for vegetative propagation of selected individuals for seedling production. This last scenario thus assumes GS under a forward selection scheme, which is possible in black spruce [1].

## Results

Genotypic and phenotypic information was gathered from a total of 734 25-year-old black spruce trees planted in two different forest environments, and representing an advanced-generation breeding population of full-sib families from crosses involving parents from nine Canadian provenances and one from Maine in the U.S.A. Population structure was found to be weak, in congruence with previous genetic studies relying on molecular markers and implicating populations from eastern Canada [52, 53, 57]. Indeed, spectral decomposition of the genomic relationship matrix [15, 25] according to the geographic distribution of origins revealed that about five percent of the variation was captured by the first eigenvector. We concluded that the genetic and genomic relationship matrices were sufficient to capture relatedness among individuals, and as such population structure was not considered in subsequent GS analyses.

### High accuracy of GS models in combined-site analyses

When the two test sites were simultaneously considered, with all markers and independent trees for validation, the accuracy of the GS models was high for all traits assessed. Moreover, these accuracy estimates were similar to those of the polygenic models using pedigree and phenotypic information (Table 2). Differences in accuracy between the different phenotypic traits for both the GS and polygenic models were also minor. Larger differences were found for predictive ability where the most accurate predictions were recorded for height growth. The accuracies largely mirrored the trends in individual trait heritability (Table 1), where high estimates of genetic control were observed for MFA (*h* ^*2*^ = 0.74) and height growth (h^2^ = 0.68), and somewhat weaker values observed for DBH and wood density (*h* ^*2*^ = 0.57 and *h* ^*2*^ = 0.41, respectively).

**Table 1:** Variance component estimates from genomic selection analyses, combined-site and single-site analyses. “Pedigree” indicates variance component estimation based on pedigree and phenotypic information, and “Markers” indicate SNP information from all 4,993 SNPs

Trait		Model	*σ* ^2^_a_^a^	*V*_A_^b^	*σ* ^2^_u_^c^	*σ* ^2^_e_^d^	*h* ^2^_i_^e^
Wood density	Combined sites	Pedigree	-	-	597.73	870.01	0.41
		Markers	0.29	551.46	-	878.19	0.39
	Site 1	Pedigree	-	-	973.94	1407.01	0.41
		Markers	0.50	957.04	-	1398.98	0.41
	Site 2	Pedigree	-	-	653.68	216.32	0.75
		Markers	0.27	523.71	-	291.00	0.64
Diameter (DBH)	Combined sites	Pedigree	-	-	126.42	93.69	0.57
		Markers	2.9E -2	55.88	-	135.02	0.29
	Site 1	Pedigree	-	-	123.22	129.34	0.49
		Markers	0.04	68.09	-	164.37	0.29
	Site 2	Pedigree	-	-	143.65	54.22	0.73
		Markers	0.03	60.26	-	106.18	0.36
Height	Combined sites	Pedigree	-	-	3503.73	1613.54	0.68
		Markers	0.97	1851.53	-	2514.81	0.42
	Site 1	Pedigree	-	-	3232.04	1623.81	0.67
		Markers	1.14	2182.23	-	2227.79	0.49
	Site 2	Pedigree	-	-	4494.96	1222.76	0.79
		Markers	1.32	2530.77	-	2341.31	0.52
Microfibril angle	Combined sites	Pedigree	-	-	0.14	4.8E -2	0.74
		Markers	3.5E -5	6.7E -2	-	8.8E -2	0.43
	Site 1	Pedigree	-	-	0.15	5.5E -2	0.73
		Markers	4.5E -5	8.6E -2	-	9.2E -2	0.48
	Site 2	Pedigree	-	-	0.15	3.1E -2	0.83
		Markers	4.3E -5	8.2E -2	-	7.2E -2	0.53

^a^ *σ* ^2^_a_ is the additive genetic variance explained by marker loci

^b^ *V*_A_ is the additive genetic variance based on markers was estimated as ${V}_A=2{\sigma}_a^2{\displaystyle {\sum}_{i=1}^k}{p}_k{q}_k.$

^c^ *σ* ^2^_u_ is the polygenic variance estimated based on pedigree

^d^ *σ* ^2^_e_ is the residual variance

^e^ *h* ^2^i is the individual trait heritability

In terms of genetic gains, the model containing trees from both sites resulted in the largest gain predictions (Table 2). The gain ratio, or in other words, that from marker models versus conventional pedigree selection was high for all traits, with DBH showing the weakest ratio (below 90%). Gain ratios above 100% were obtained for wood traits, indicating that marker-based models would allow for better gains to be obtained than pedigree-based models. When considering the potential time saved for breeding based on GS, the annual gain ratios for marker-based models versus conventional selection increased considerably to being around 3. This is because GS is conducted without field testing and thus, avoids the delays associated with tree growing and assessing phenotypic trait variation from mature trees.

**Table 2:** Genomic selection analyses based on all marker information (4,993 SNPs) and following different schemes of model building and application. “Pedigree” indicates that only pedigree information was used for prediction after model calibrations, which is also referred to as the conventional breeding approach in the body of the text; “Markers” indicates that SNP information was used for prediction. All results are from cross-validations using 10 replicates on randomly-selected trees not included in model fitting, but from the same families used to fit models. The first scheme considered all 734 trees from the two sites combined. Models in other schemes were trained on one site only and whether applied on the same or on the other site, respectively. For comparative purposes, the “Site_mean” scheme represents the mean of 5 models run on 359 trees corresponding to the mean number of trees per site. Model accuracy is the correlation between the cross-validated estimated breeding value (using independent sets of trees) and the “true” reference breeding value. The predictive ability is the correlation between the predicted and the actual phenotypes. Genetic gains are given in absolute values and percentages are given in brackets. Gain estimates are based on predicted phenotypes and a selection intensity of 5%. Annual gain estimates were based on assumptions of a conventional breeding cycle length of 28 years for pedigree selection (“Pedigree”), and a shortened cycle length of 9 years for selection with markers (“Markers”), with 4 years for crosses and production of seedlots that are full-siblings to the training population, followed by 1 year for selection of individuals using markers and genomic selection models, and 4 years for vegetative propagation of selected individuals for seedling production

Trait	GS scenario	Accuracy (error)		Predictive ability (error)		Gain^a^ (percent)		Gain ratio	Gain per year		Gain per year ratio
		Pedigree	Markers	Pedigree	Markers	Pedigree	Markers	M/P^b^	Pedigree	Markers	M/P^b^
Wood density	Combined sites	0.89 (0.03)	0.84 (0.02)	0.45 (0.09)	0.49 (0.07)	34.74 (0.08)	35.63 (0.09)	1.03	1.24	3.96	3.19
	Site1	0.80 (0.08)	0.77 (0.06)	0.34 (0.16)	0.38 (0.12)	35.90 (0.09)	31.87 (0.08)	0.89	1.28	3.54	2.77
	Site2	0.88 (0.05)	0.82 (0.10)	0.52 (0.15)	0.56 (0.12)	26.25 (0.06)	29.48 (0.07)	1.12	0.94	3.28	3.49
	Site_mean	0.85 (0.05)	0.81 (0.05)	0.42 (0.18)	0.43 (0.16)	30.15 (0.07)	28.55 (0.07)	0.95	1.08	3.17	2.94
	Site1 → 2	0.79 (0.05)	0.77 (0.08)	0.42 (0.16)	0.44 (0.19)	24.60 (0.06)	21.47 (0.05)	0.87	0.88	2.39	2.72
	Site2 → 1	0.85 (0.04)	0.80 (0.08)	0.36 (0.13)	0.39 (0.11)	40.02 (0.10)	45.46 (0.11)	1.14	1.43	5.05	3.53
Height	Combined sites	0.88 (0.03)	0.86 (0.03)	0.56 (0.08)	0.57 (0.07)	105.17 (0.13)	104.57 (0.13)	0.99	3.76	11.62	3.09
	Site1	0.84 (0.05)	0.81 (0.04)	0.51 (0.11)	0.51 (0.11)	53.95 (0.07)	52.17 (0.07)	0.97	1.93	5.8	3.01
	Site2	0.88 (0.02)	0.85 (0.03)	0.58 (0.09)	0.58 (0.12)	82.89 (0.10)	78.69 (0.10)	0.95	2.96	8.74	2.95
	Site_mean	0.85 (0.05)	0.83 (0.05)	0.55 (0.12)	0.55 (0.12)	87.00 (0.11)	84.56 (0.11)	0.97	3.11	9.4	3.02
	Site1 → 2	0.84 (0.03)	0.83 (0.04)	0.56 (0.12)	0.56 (0.13)	75.93 (0.09)	74.56 (0.09)	0.98	2.71	8.28	3.06
	Site2 → 1	0.85 (0.04)	0.84 (0.03)	0.51 (0.11)	0.52 (0.11)	63.67 (0.08)	62.31 (0.08)	0.98	2.27	6.92	3.05
Diameter (DBH)	Combined sites	0.86 (0.03)	0.83 (0.04)	0.45 (0.06)	0.43 (0.07)	16.65 (0.14)	14.81 (0.13)	0.89	0.59	1.65	2.80
	Site1	0.76 (0.08)	0.74 (0.08)	0.38 (0.15)	0.34 (0.15)	12.09 (0.10)	10.15 (0.09)	0.84	0.43	1.13	2.63
	Site2	0.87 (0.02)	0.82 (0.04)	0.53 (0.05)	0.48 (0.08)	14.70 (0.13)	13.18 (0.12)	0.90	0.53	1.46	2.75
	Site_mean	0.82 (0.05)	0.79 (0.06)	0.45 (0.09)	0.42 (0.11)	13.44 (0.12)	11.84 (0.10)	0.88	0.48	1.32	2.75
	Site1 → 2	0.76 (0.05)	0.75 (0.05)	0.43 (0.09)	0.43 (0.10)	10.99 (0.10)	9.65 (0.08)	0.88	0.39	1.07	2.74
	Site2 → 1	0.80 (0.05)	0.78 (0.05)	0.33 (0.13)	0.34 (0.12)	16.09 (0.14)	13.75 (0.12)	0.85	0.57	1.53	2.68
Microfibril angle^c^	Combined sites	0.88 (0.03)	0.84 (0.04)	0.51 (0.11)	0.51 (0.10)	−2.71 (−0.15)	−2.78 (−0.15)	1.02	−0.10	−0.31	3.10
	Site1	0.83 (0.06)	0.79 (0.04)	0.47 (0.15)	0.45 (0.13)	−2.47 (−0.12)	−2.29 (−0.11)	0.92	−0.09	−0.25	2.78
	Site2	0.86 (0.04)	0.82 (0.04)	0.54 (0.16)	0.52 (0.15)	−1.72 (−0.11)	−1.80 (−0.12)	1.09	−0.06	−0.20	3.33
	Site_mean	0.84 (0.06)	0.80 (0.05)	0.48 (0.15)	0.48 (0.14)	−1.84 (−0.11)	−1.80 (−0.11)	1.00	−0.07	−0.20	2.86
	Site1 → 2	0.80 (0.03)	0.76 (0.04)	0.48 (0.13)	0.47 (0.12)	−2.05 (−0.13)	−1.95 (−0.12)	0.92	−0.07	−0.22	3.14
	Site2 → 1	0.84 (0.04)	0.81 (0.04)	0.44 (0.13)	0.45 (0.11)	−2.14 (−0.10)	−2.16 (−0.10)	1.01	−0.08	−0.24	3.00

^a^Genetic gains are given in absolute values; units are kg/m^3^ for wood density, cm for height, mm for diameter and degrees for microfibril angle

^b^M/P, markers to pedigree gain ratio

^c^Negative genetic gain for MFA indicates trait improvement

### Between-site differences in genomic selection models in black spruce

In order to assess the extent of the genotype-by-environment interaction, and to evaluate if GS model accuracy is affected by sites, we constructed and validated GS models for each of the two different sites (Table 2). Accuracy estimates of models built using data from one site only were slightly inferior compared with accuracies obtained with the combined-site analyses, especially for site 1. Accuracy estimates of models developed with data collected on one site and validated with data collected from the second site were high, and only marginally lower than estimates for validations carried out within the same site. These results indicate that genotype-by-environment interaction is low, and that GS models can most likely be applied over a large range of sites without the need for increased tree sampling for independent model construction, given that the two sites in the current study represented quite contrasting environmental conditions.

The relatively good overall model accuracies obtained by applying models developed on one site and applied to the second one does not mask important within-site environmental differences. Field records identified more trait variation and vegetation competition on site 1 (Matapedia, warmer site) compared with site 2 (Robidoux, colder site), which led to lower heritability estimates for site 1 (Table 1). Additionally, a smaller number of trees were available for site 1 compared to site 2 (333 and 401 trees analysed, respectively). Hence, GS models trained on site 2 and validated on site 1 or site 2 were marginally more accurate, which was especially true for wood density and diameter. The same trend was observed for prediction models based on pedigree and phenotypic information.

### Relatedness

Models built with a half-sib structure led to a large decrease in accuracy, predictability and genetic gain (Table 3). Although model validation was set up equally for all traits, accuracy varied much more among traits than when full-sib models were applied. The loss of model quality was most pronounced for MFA, where accuracy was only half. When cross-validation was conducted with completely unrelated progeny from different provenances and families (whether with full-sib or half-sib structure), model accuracy dropped virtually to zero and was associated with high error rates (results not shown).

**Table 3:** Accuracies of genomic selection models based on half-sib families using all 4,993 markers in a combined-site analysis, thus removing full-sib family linkage. Pedigree indicates that only pedigree information was used for predictions after model calibrations; markers indicates that SNP information was used. The predictive ability is the correlation between the predicted and the actual phenotypes. Genetic gains are given in absolute values and percentages are given in brackets. Gain estimates are based on predicted phenotypes and a selection intensity of 5%. Annual gain estimates were based on assumptions of a conventional breeding cycle length of 28 years for pedigree selection (“Pedigree”), and a shortened cycle length of 9 years for selection with markers (“Markers”), with 4 years for crosses and production of seedlots that are full-siblings to the training population, followed by 1 year for selection of individuals using markers and genomic selection models, and 4 years for vegetative propagation of selected individuals for seedling production

Trait	Accuracy (error)		Predictive ability (error)		Gain^a^ (percent)		Gain ratio	Gain per year		Gain per year ratio
	Pedigree	Markers	Pedigree	Markers	Pedigree	Markers	M/P^b^	Pedigree	Markers	M/P^b^
Wood density	0.77 (0.14)	0.65 (0.18)	0.38 (0.16)	0.37 (0.15)	26.48 (0.06)	26.33 (0.06)	0.99	0.95	2.93	3.08
Height	0.80 (0.12)	0.76 (0.13)	0.50 (0.19)	0.49 (0.18)	78.59 (0.10)	76.70 (0.10)	0.98	2.81	8.52	3.03
Diameter (DBH)	0.64 (0.17)	0.63 (0.17)	0.30 (0.20)	0.31 (0.21)	14.41 (0.13)	12.19 (0.11)	0.85	0.51	1.35	2.65
Microfibril angle^c^	0.40 (0.41)	0.42 (0.28)	0.18 (0.32)	0.23 (0.25)	−2.01 (−0.11)	−1.97 (−0.11)	1.00	−0.07	−0.22	3.14

^a^Genetic gains are given in absolute values; units are kg/m^3^ for wood density, cm for height, mm for diameter and degrees for microfibril angle

^b^M/P, markers to pedigree gain ratio

^c^Negative genetic gain for MFA indicates trait improvement

### Marker subsets inform on the nature of causative linkage disequilibrium

**Fig. 1:**
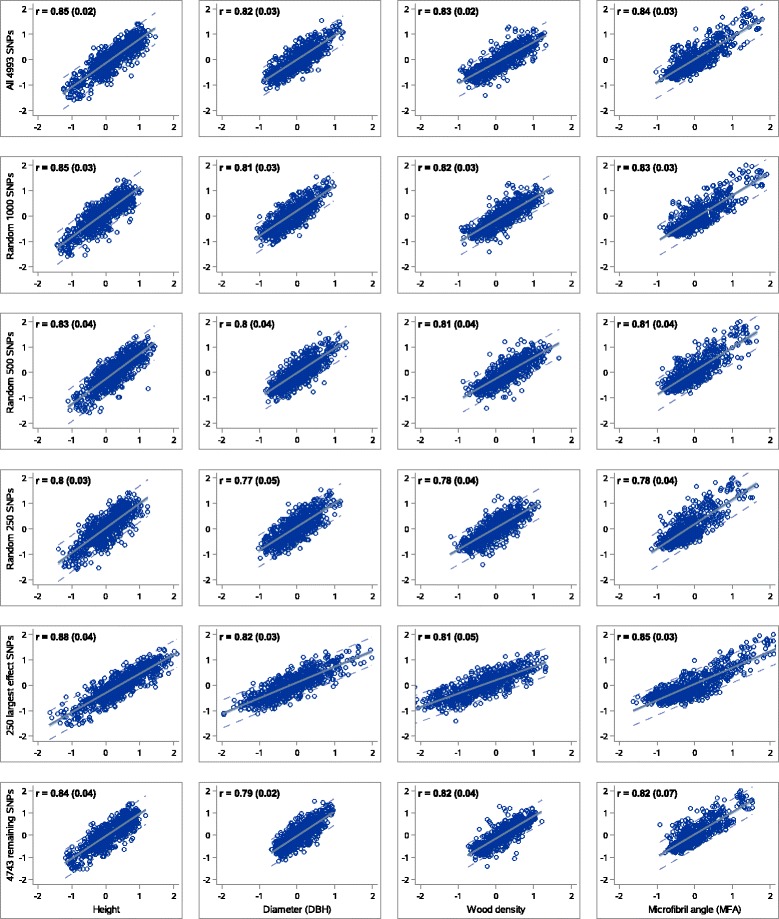
Accuracy of genomic selection models with reduced marker density. Accuracy estimates for subsets of markers are shown by correlations between the genomic-predicted breeding values (x-axis) and the true breeding values (y-axis) for tree height, diameter at breast height (DBH), wood density, and microfibril angle. Associated errors of accuracy estimates are presented in brackets. On the y-axis of the fifth row of plots, largest SNPs indicate SNPs with largest absolute effects. On the y-axis of the sixth row of plots, remaining SNPs indicate the subset of SNPs without those 250 SNPs with largest absolute effects

We also investigated the effect of reduced marker sets on the accuracy of GS models. The use of fewer markers would result in significant cost reductions for genotyping, which would have some impact on the overall cost of GS, but would also impact the extent of LD that is picked up by the prediction models. Decreasing the number of markers from almost 5,000 to 1,000 randomly sampled markers did not lead to a notable reduction in the accuracy of GS models (Fig. 1). However, using less than 500 markers led to an appreciable loss in accuracy for all four traits. Figure 1 shows the accuracy plots for several scenarios, where the loss of accuracy was mostly related to less scattered correlation plots due to a range reduction of genomic-estimated breeding values (GEBV).

The modelling of 250 markers that had previously been identified to contribute the largest absolute effects in the basic model, led to accuracy estimates comparable to those of the basic model including all 4,993 markers (Fig. 1). These models also perform substantially better than models based on 250 random markers, which is again related to an increase of range in GEBV estimates (Fig. 1). However, the average MAF of largest-effect markers was also significantly higher (student t, *P* < 0.0001, for all traits) than the average MAF of remaining 4,743 markers of lower effects. Markers with the largest effects most likely retraced family linkages better, given their higher MAF and thus, higher information value. Modelling the remaining 4,743 lower-effect markers led to accuracies marginally lower than estimates for the full model.

### Linkage groups have similar effects on GS model accuracies

**Fig. 2:**
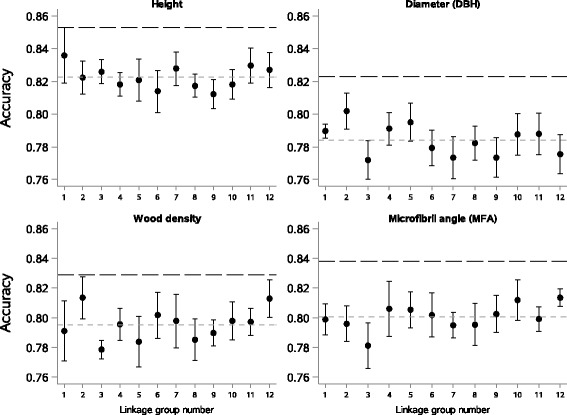
Effect of linkage group on accuracy of genomic selection models. Accuracy (*black* circles) and associated errors for models based on markers pertaining to the same individual linkage group. Dashed grey lines indicate the means of accuracy estimates of the 12 linkage groups; long-dashed black lines are the accuracies of models combining all markers of known map positions (2928 markers, see Methods) and spanning the entire genome

In a further analysis, we investigated the effect of the genomic location of markers of GS models for the different traits. Therefore, we used markers of genes for which a set of 2928 homologs have been recently mapped to the white spruce genome [48], which has been previously shown to be highly syntenic et collinear to the black spruce genome [51]. GS analyses considering sets of markers delimited according to linkage groups showed some differences in accuracy estimates from one chromosome to another, but these differences were not statistically significant (Fig. 2). At this marker resolution, we were thus unable to identify particular linkage groups that would control more for a specific trait. Overall, the accuracies obtained for the four traits were lower than those of models based on all markers, but they were slightly higher than estimates for models based on an equivalently reduced number of markers but sampled randomly across the entire genome (see Fig. 1, random 250 SNPs). These results suggest that relatedness is a likely strong contributing factor to the high prediction accuracy of GS models.

### Sample sizes used to build genomic selection models

**Fig. 3:**
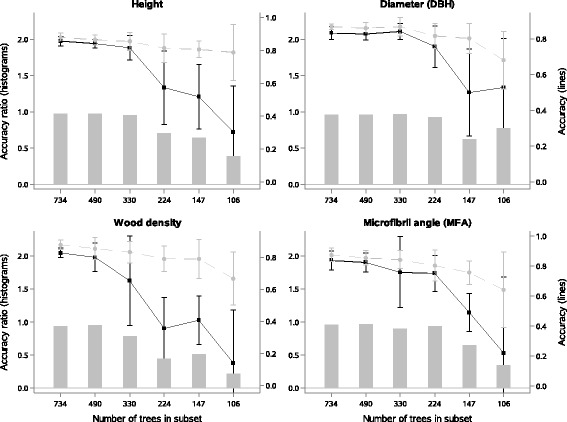
Accuracy of genomic selection models obtained using subsets of trees. Accuracy estimates for pedigree-based models (light grey line) and marker-based models (dark line), as well as their ratio (histograms). Estimates are given with their associated standard errors

The effect of sample size used for GS model training was investigated. Starting with the full set of 734 sampled trees from both sites, we randomly removed trees and evaluated model quality for various subsets. As expected, accuracy of the GS models from the combined-site analysis generally decreased when fewer trees were used (Fig. 3). The reduction was much more important in marker-based models compared with pedigree-based models, leading to decay in the accuracy ratio. When considering less than a quarter of the trees initially present in the training set, model accuracy decreased by 50% on average. Sample sets with 330 or more trees showed comparable or only marginally inferior model accuracy compared with the full sample set, picking up well existing LD and relatedness. However, when training models with this number of trees, the accuracy of models was associated with larger errors for wood quality traits, especially for wood density where accuracy degraded faster and more irregularly in both marker-based and pedigree-based models.

**Fig. 4:**
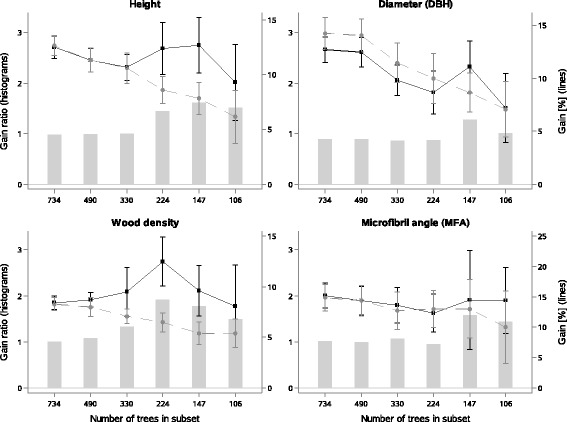
Predicted genetic gain using subsets of trees to build pedigree-based models (light grey line) and marker-based models (dark line), and the corresponding coefficient of variation (error bars). The ratio of marker- to pedigree-predicted genetic gain is presented by histograms. Gain estimates are based on predicted phenotypes and a selection intensity of 5%

Similarly, errors of predicted genetic gain based on the use of GS models increased for sample sets of 330 trees or less. This was especially true for tree height and wood density where the average predicted genetic gain of marker-based models even increased slightly, when models were based on low numbers of individuals (Fig. 4). Together with the important loss of accuracy for both traits (Fig. 3), this result highlights the value of a large training set leading to precise accuracy estimates from GS models in order to make well-grounded selection decisions.

## Discussion

### Accuracy of genomic selection models with complete information

This study clearly shows that medium-dense marker panels with several thousand markers well distributed over the genome can be effectively used in GS to predict additive breeding values in advanced-generation tree breeding programs, echoing previous results in similar settings [21, 24, 25, 33]. The accuracy of the GS models obtained with the present black spruce breeding population was high for both growth and wood quality traits, reaching values of approximately 0.8 when using several hundred trees to build the GS models. Accuracy estimates from GS models were comparable with their pedigree-based counterparts, and were superior to results obtained in white spruce for a population of full-sib families [25]. These findings suggest that the current marker panel was marginally more efficient than the pedigree information in retracing family linkages (see below), especially if some errors affected pedigree information. These results lead to the conclusion that GS can efficiently be applied for this boreal conifer species in advanced-breeding programs and highly structured populations of full-sib families, resulting in much higher gains per year than conventional selection. Moreover, applying a forward GS scheme at an early stage appears possible in black spruce, given that it inherently displays a high propensity for vegetative propagation at an early age, as seen for other spruces [1].

The present accuracy estimates were somewhat higher than estimates previously obtained for similar traits in full-sib families of white spruce of similar age in a comparable study [25], and they were considerably higher than accuracy estimates obtained for loblolly pine [22]. Following the simulation results of Grattapaglia and Resende [32] and the parameters of the present study, one would expect accuracy estimates ranging between 0.7 and 0.8 for genomic models relying on 2 to 3 markers per cM, with an effective population size close to 30, as well as a training population set somewhat below 1,000 individuals. The present estimates are on the upper limit or surpass these expectations, likely because of the high heritabilities observed in the present black spruce field trial for these traits.

Overall, the accuracy estimates only showed minor differences among traits. Model quality for tree diameter was somewhat lower than that for tree height, which is congruent with earlier reports [24, 25]. This is most likely related to the higher heritability of tree height compared to diameter, as often noted in conifers [25, 27, 58].

### Genotype-by-environment interaction

The genotype-by-environment interaction was low. Models calibrated on one site led to good predictions of GEBVs on the other site, indicating low genotype-by-environment interactions in this test despite the contrasting site conditions and the large geographic distribution of parental trees used to produce the full-sib families. Low genotype-by-environment interactions were also previously reported from provenance-progeny tests replicated on multiple sites in Québec [59, 60]. Similar observations of good transfer of model accuracy among distant sites from two large breeding zones in Québec were also reported for white spruce for both half-sib and full-sib GS models [25, 26]. These results contrast with reports on hybrid spruce from western Canada [27] and loblolly pine from the southeastern United States [33], where the need to recalibrate GS models in each breeding zone was shown.

Boreal spruces in eastern Canada, such as black and white spruces, are reforested on a geographically restricted land base compared to the extent of their natural distribution, at the southern edge of their natural range where the commercial forest is mostly located. Based on provenance-progeny tests targeting these reforestation areas, reduced genotype-by-environment interaction was noted and the reforestation sites have been split into only a few large breeding zones [40, 60]. Furthermore, little phylogeographic structure has been reported in the province of Québec and its vicinity for black spruce [52, 53, 61, 62] or white spruce [63], indicating a homogenous historical genetic background. Little among-population differentiation has also been observed with various molecular markers, indicating limited population structuring and reflecting the recent post-glacial recolonization in eastern Canada [52, 53, 57, 64]. Furthermore, the parental trees employed in the present black spruce advanced-breeding populations were first-generation superior trees selected in provenance tests assembling multiple provenances, well scattered geographically beyond single breeding zones and performing well on multiple sites. Therefore, their genetic background may have been indirectly selected toward generalists bearing a plastic adaptability to local conditions. This study shows that models can be applied to different sites or be built by pooling data from different sites without significantly compromising accuracy. From a practical point of view, it also means that fewer trees are necessary to train and obtain models with good accuracy, thus reducing phenotyping and genotyping costs for GS model development.

### The effect of size of model training set on genomic selection accuracy

The size of the dataset employed to train models has previously been shown to have a large effect on the accuracy of GS models [32]. In advanced-breeding populations with small effective population size, we hypothesized that a smaller number of samples per progeny should be sufficient to obtain good model accuracy. Using resampling, Perron et al. [65] reported that optimal subgroups should include 6 to 8 trees per family and site in order to precisely estimate genetic parameters for wood density and growth in an open-pollinated black spruce test. Our current finds concur with those of Perron et al. [65], as a sizeable loss in accuracy was only observed when the training sets were less than 330 trees, corresponding to a minimum of about 4 trees per site and full-sib family in the present context. One interesting observation was that marker-based models had a tendency to loose accuracy more quickly than their pedigree-based counterparts. From a practical point of view, a smaller number of trees necessary to train and obtain models with good accuracy will help reduce the genotyping and phenotyping costs for GS model development.

### Genomic selection model accuracy and level of family structure

Genomic selection models constructed using data from a subset of half-sibs from the same test resulted in much lower and more variable accuracies. However, models built with half-sibs had higher accuracies than those from previous reports similarly derived from half-sib families of eastern white spruce or western hybrid spruce in Canada [26, 28]. The difference is likely due to the low effective population size of half-sibs in the present study, leading to a higher level of relatedness among trees in the training and validation sets compared to true open-pollinated families where a large number of mostly unrelated pollen donors intervene and greatly increase the effective population size [26].

The shift in accuracy and predicted genetic gain observed between using half-sib versus full-sib sets further highlights that GS is most efficiently applied in more structured populations where relatedness and LD are higher, which necessitates less genome coverage to attain high prediction accuracy. Similar observations were reported by Beaulieu et al. [25, 26] and Zapata-Valenzula et al. [21]. Below, we discuss additional evidence to support this interpretation.

### A limited role for short-range LD in genomic prediction

The main obstacles for the application of MAS in largely undomesticated populations of conifers are their large genome sizes often exceeding 20 Gbp [66] and their low LD [35, 36], which in turn would require very high genome coverage in order to pick-up short-range marker-QTL LD and make accurate predictions; this, besides the multigenic control of most relevant traits was already identified as a drawback in association studies [5–7] where single markers explained only a low percent of variance. In this context, the role of short-range marker-QTL LD in the accuracy of GS models obtained with moderate genome coverage appears negligible. In the present study, a density of approximately 2.7 markers/cM was used, which resulted in GS model accuracy roughly equivalent to that from pedigree-based models. The same trend was also observed in previous studies [25, 26]. GS model accuracy decreased significantly when using half-sibs instead of full-sibs, which is consistent with the trend seen from other studies displaying similar genome coverage [25–27]. Using only markers with large effects resulted in marginally better model accuracies compared to those obtained with same number of markers randomly picked. However, we showed that this aspect is entangled with higher average MAF values for these markers. Also, the non-significant differences among model accuracies obtained with markers from different chromosomes likely indicates that limited short-range marker-QTL LD could be traced. Altogether, these observations point to relatedness and an increasing size of un-recombined chromosome blocks as the main drivers of GS model accuracy. Relatedness and the ability to retrace family linkages should be seen as the key factors for the high accuracies obtained, given that restricting GS model building with markers from single chromosomes led to somewhat higher accuracies compared to models relying on an equivalent random sample of markers covering all 12 spruce chromosomes. This trend is further supported by the fact that increasing the number of markers used to build GS models tenfold (from 500 to 4,993 markers) only led to incremental, though useful, improvements in model accuracy.

These observations are further supported by the fact that when GS models were applied to trees from families not included during model building, little or no accuracy was obtained, further confirming the limited role of short-range LD in the high accuracies obtained when using full-sibs. These results are not surprising given that black spruce and most conifers are essentially undomesticated, outbreed, wind-pollinated organisms with large effective population sizes, hence lacking population-wide LD. For instance, LD decays rather rapidly in natural populations of spruces and pines, usually well within gene limits and in many cases, within a few hundred base pairs, which is a likely consequence of historically large effective population sizes [35, 36]. Consequently, our results and those of others (e.g. [22, 23]) suggest that genomic prediction may not be possible for unrelated individuals at current marker densities.

A corollary is that obtaining GS model accuracy beyond that of pedigree-based models would likely necessitate increasing genome coverage by a factor of at least 10X to 100X of that used herein, resulting in the use of a very large number of markers, likely in the hundreds of thousands. Simulations and historical data in cattle led to the conclusion that 50k markers would allow for the capture of causal loci within breeds, but 300k markers would be needed for accurate prediction across breeds [67]. A similar trend is emerging for crop plants, with the preparation of genotyping arrays containing over half a million SNPs (e.g. McCouch C. et al., International Rice Consortium, in preparation). Furthermore, based on simulations, Grattapaglia and Resende [32] showed that increasing genome coverage tenfold from 1–2 markers/cM to 10–20 markers/cM only asymptotically improves GS model accuracy.

The fact that slightly higher GS model accuracy was obtained with models using only markers with largest effects (top 250 out of 4,993 markers), compared to models estimated with all markers, could imply that part of the short-range LD can be picked-up by the GS models when a reduced numbers of large-effect markers are employed. However, there could be confounding factors, such as the *a priori* information value of markers. Indeed, a significantly higher average value of minimum allele frequency (MAF) was observed for markers with largest effects. Such markers could track family linkages more effectively than random markers, especially when small numbers of markers are used. Also, the marginally higher accuracy achieved with markers located on a specific chromosome compared to same number of markers but spread over the entire genome indicate that a better tracking of family linkages is achieved when a small number of markers are located on the same chromosome. Thus, this factor could also be potentially useful to reduce genotyping and GS costs in highly structured populations when the genome location of markers is known.

## Conclusions

The results of the present study on black spruce indicate that, at least in the short term, GS holds substantial promises for efficient application in populations of small effective size, such as advanced-breeding populations. With the scale of marker densities usually employed (at a rate of a few markers per cM), the overall short-range LD between markers and causal loci is likely not sufficiently well captured to make GS efficient in largely unstructured natural populations of conifers or between unrelated breeding populations. In small and structured populations, we show that prediction accuracy is null when relatedness between training and testing data sets is absent. Marker densities of one or several orders of magnitude higher would likely be necessary to improve accuracy in such conditions. Thus, high relatedness between individuals appears to be a prerequisite to obtain highly accurate GEBVs.

From an applied point of view, lowering marker densities may be feasible without major loss in accuracy in order to reduce genotyping costs. Information relative to relatedness would be more precise when markers are located on a single chromosome instead of spreading an equivalent number of markers over the whole genome, and with using markers with highest MAF. These approaches may be considered when a reduced genotyping assay is needed.

Based on the present results, the GS application with highest potential for spruce breeders would be to select with high accuracy superior individuals within a group of full-sib families. This would effectively increase the relatedness as well as the size of unrecombined chromosome blocks generated by controlled crossing in a small breeding population. One obvious beneficial implementation of GS in such a context would be to repeat the controlled crosses that were used to build the GS models to generate much larger full-sib families and thus, apply higher selection intensities and obtain larger genetic gains. Future studies also need to evaluate to which extent genomic selection models developed for the present generation could be applied to the next generation of progeny, as recombination should break up some of the established linkage [23]. However, given the good accuracy of GS models that we obtained when considering individuals sharing only one parent, we hypothesize that predictions in a following generation may be relatively accurate when sharing the same parental material. Also, because family linkages could be efficiently traced with genomic profiles, polycross strategies could likely be used without losing significantly on prediction accuracy, where male pollen donors are mixed so to reduce the cost of crosses. Such screening of larger families from polycross would make it possible to increase selection intensity and the ensuing genetic gain in a cost-effective fashion, especially for traits such as wood quality parameters or pest resistance, which are expensive and cumbersome to assess on large test populations. Thus, candidate individuals for selection would only have to be genotyped at the early seedling stage and those having the highest GEBVs predicted using the available GS models would be selected.

For species amenable to vegetative propagation and for organizations having access to somatic embryogenesis and/or rooted cutting facilities, as it is the case in eastern Canada for spruces, individuals identified with GS at the early seedling stage could also be propagated and mass-produced for reforestation programs within only a few years [1]. With such a forward selection scheme, the breeding and production cycles could be significantly reduced and gain per time unit would be multiplied by a factor of 3 (Table 2). Other schemes based on sexual reproduction could be deployed, such as top-grafting of selected progeny in previous generation seed orchards followed by polymix crossing, which would facilitate the production of genetically improved seeds with minimal delays. At the same time and by shortening quite drastically the breeding cycles for slow-growing species such as temperate and boreal conifers, the use of GS tools should result in more flexibility to tree breeders, which appears especially important in the context of rapid environmental changes and evolution of wood products markets.

## Abbreviations

cM: Centimorgan
EBV: Estimated breeding value
GEBV: Genomic estimated breeding value
GS: Genomic selection
LD: Linkage disequilibrium
MAF: Minor allele frequency
MAS: Marker-assisted selection
MFA: Cellulose microfibril angle in the secondary cell wall
QTL: Quantitative trait locus
SNP: Single nucleotide polymorphism

## References

[1] Park Y-S, Beaulieu J, Bousquet J, Park YS, Bonga JM, Moon H-K (2016). Multi-varietal forestry integrating genomic selection and somatic embryogenesis. Vegetative Propagation of Forest Trees.

[2] White TL, Neale DB, Adams WT (2007). Forest Genetics.

[3] Burdon RD, Wilcox PL, Plomion C, Bousquet J, Kole C (2011). Integration of molecular markers in breeding. Genetics, Genomics and Breeding of Conifers.

[4] Lande R, Thompson R (1990). Efficiency of marker-assisted selection in the improvement of quantitative traits. Genetics.

[5] Porth I, Klapšte J, Skyba O, Hannemann J, McKown AD, Guy RD, et al. Genome-wide association mapping for wood characteristics in *Populus* identifies an array of candidate single nucleotide polymorphisms. New Phytol. 2013;200:710–26. 10.1111/nph.12422 23889164

[6] Beaulieu J, Doerksen T, Boyle B, Clement S, Deslauriers M, Beauseigle S (2011). Association genetics of wood physical traits in the conifer white spruce and relationships with gene expression. Genetics.

[7] Gonzalez-Martinez SC, Wheeler NC, Ersoz E, Nelson CD, Neale DB (2007). Association genetics in *Pinus taeda* L. I. Wood property traits. Genetics.

[8] Holliday JA, Ritland K, Aitken SN (2010). Widespread, ecologically relevant genetic markers developed from association mapping of climate-related traits in Sitka spruce (*Picea sitchensis*). New Phytol..

[9] Prunier J, Pelgas B, Gagnon F, Desponts M, Isabel N, Beaulieu J (2013). The genomic architecture and association genetics of adaptive characters using a candidate SNP approach in boreal black spruce. BMC Genomics.

[10] Ritland K, Krutovsky KV, Tsumura Y, Pelgas B, Isabel N, Bousquet J, Plomion C, Bousquet J, Kole C (2011). Genetic mapping in conifers. Genetics, Genomics and Breeding of Conifers.

[11] Pelgas B, Bousquet J, Meirmans PG, Ritland K, Isabel N (2011). QTL mapping in white spruce: gene maps and genomic regions underlying adaptive traits across pedigrees, years and environments. BMC Genomics.

[12] Meuwissen T, Hayes B, Goddard M (2001). Prediction of total genetic value using genome-wide dense marker maps. Genetics.

[13] Grattapaglia D, Plomion C, Kirst M, Sederoff RR (2009). Genomics of growth traits in forest trees. Curr Opin Plant Biol.

[14] Hayes B, Goddard M (2010). Genome-wide association and genomic selection in animal breeding. Genome.

[15] VanRaden P (2008). Efficient methods to compute genomic predictions. J Dairy Sci.

[16] Legarra A, Robert-Granié C, Manfredi E, Elsen J-M (2008). Performance of genomic selection in mice. Genetics.

[17] Desta ZA, Ortiz R (2014). Genomic selection: genome-wide prediction in plant improvement. Trends Plant Sci.

[18] Jannink JL, Lorenz AJ, Iwata H (2011). Genomic selection in plant breeding: from theory to practice. Brief Funct Genomics.

[19] Heffner EL, Sorrells ME, Jannink J-L (2009). Genomic selection for crop improvement. Crop Sci.

[20] Resende MDV, Resende MFR, Sansaloni CP, Petroli CD, Missiaggia AA, Aguiar AM (2012). Genomic selection for growth and wood quality in *Eucalyptus*: capturing the missing heritability and accelerating breeding for complex traits in forest trees. New Phytol..

[21] Zapata-Valenzuela J, Isik F, Maltecca C, Wegrzyn J, Neale D, McKeand S (2012). SNP markers trace familial linkages in a cloned population of *Pinus taeda*—prospects for genomic selection. Tree Genet. Genomes.

[22] Resende MFR, Muñoz P, Resende MDV, Garrick DJ, Fernando RL, Davis JM (2012). Accuracy of genomic selection methods in a standard dataset of Loblolly pine (*Pinus taeda* L.). Genetics.

[23] Bartholomé J, Van Heerwaarden J, Isik F, Boury C, Vidal M, Plomion C (2016). Performance of genomic prediction within and across generations in maritime pine. BMC Genomics.

[24] Isik F, Bartholomé J, Farjat A, Chancerel E, Raffin A, Sanchez L (2016). Genomic selection in maritime pine. Plant Sci.

[25] Beaulieu J, Doerksen TK, MacKay J, Rainville A, Bousquet J (2014). Genomic selection accuracies within and between environments and small breeding groups in white spruce. BMC Genomics.

[26] Beaulieu J, Doerksen T, Clément S, MacKay J, Bousquet J (2014). Accuracy of genomic selection models in a large population of open-pollinated families in white spruce. Heredity.

[27] Gamal El-Dien O, Ratcliffe B, Klápště J, Chen C, Porth I, El-Kassaby YA (2015). Prediction accuracies for growth and wood attributes of interior spruce in space using genotyping-by-sequencing. BMC Genomics.

[28] Ratcliffe B, Gamal El-Dien O, Klápště J, Porth I, Chen C, Jaquish B (2015). A comparison of genomic selection models across time in interior spruce (*Picea engelmannii × glauca*) using unordered SNP imputation methods. Heredity.

[29] De La Torre A, Birol I, Bousquet J, Ingvarsson P, Jansson S, Jones SJ (2015). Insights into conifer giga-genomes. Plant Physiol.

[30] Mullin TJ, Andersson B, Bastien J, Beaulieu J, Burdon R, Dvorak W, Plomion C, Bousquet J, Kole C (2011). Economic importance, breeding objectives and achievements. Genetics, Genomics and Breeding of Conifers.

[31] Kremer A (1992). Predictions of age-age correlations of total height based on serial correlations between height increments in Maritime pine (*Pinus pinaster* Ait.). Theor Appl Genet.

[32] Grattapaglia D, Resende MDV (2011). Genomic selection in forest tree breeding. Tree Genet. Genomes.

[33] Resende M, Munoz P, Acosta J, Peter G, Davis J, Grattapaglia D (2012). Accelerating the domestication of trees using genomic selection: accuracy of prediction models across ages and environments. New Phytol..

[34] Isik F (2014). Genomic selection in forest tree breeding: the concept and an outlook to the future. New For..

[35] Pavy N, Namroud M, Gagnon F, Isabel N, Bousquet J (2012). The heterogeneous levels of linkage disequilibrium in white spruce genes and comparative analysis with other conifers. Heredity.

[36] Neale DB, Savolainen O (2004). Association genetics of complex traits in conifers. Trends Plant Sci.

[37] Perry DJ, Bousquet J (2001). Genetic diversity and mating system of post-fire and post-harvest black spruce: an investigation using codominant sequence-tagged-site (STS) markers. Can J For Res.

[38] Hill WG (1981). Estimation of effective population size from data on linkage disequilibrium. Genet Res.

[39] Pavy N, Gagnon F, Deschênes A, Boyle B, Beaulieu J, Bousquet J (2016). Development of highly reliable *in silico* SNP resource and genotyping assay from exome capture and sequencing: an example from black spruce (*Picea mariana*). Mol Ecol Resour.

[40] Beaulieu J, Corriveau A, Daoust G. Phenotypic stability and delineation of black spruce breeding zones in Québec: Natural Ressources Canada, Canadian Forest Service, Laurentian Forestry Centre, Information Report Lau-X-85E; 1989.

[41] Pavy N, Gagnon F, Rigault P, Blais S, Deschênes A, Boyle B (2013). Development of high-density SNP genotyping arrays for white spruce (*Picea glauca*) and transferability to subtropical and nordic congeners. Mol Ecol Resour.

[42] Ukrainetz NK, Kang KY, Aitken SN, Stoehr M, Mansfield SD (2008). Heritability and phenotypic and genetic correlations of coastal Douglas-fir (*Pseudotsuga menziesii*) wood quality traits. Can J For Res.

[43] Jackman SD, Warren RL, Gibb EA, Vandervalk BP, Mohamadi H, Chu J (2016). Organellar genomes of white spruce (*Picea glauca*): assembly and annotation. Genome Biol Evol.

[44] Warren RL, Keeling CI, Yuen MMS, Raymond A, Taylor GA, Vandervalk BP (2015). Improved white spruce (*Picea glauca*) genome assemblies and annotation of large gene families of conifer terpenoid and phenolic defense metabolism. Plant J.

[45] Birol I, Raymond A, Jackman SD, Pleasance S, Coope R, Taylor GA (2013). Assembling the 20 Gb white spruce (*Picea glauca*) genome from whole-genome shotgun sequencing data. Bioinformatics.

[46] Nystedt B, Street NR, Wetterbom A, Zuccolo A, Lin Y-C, Scofield DG (2013). The Norway spruce genome sequence and conifer genome evolution. Nature.

[47] Pavy N, Pelgas B, Laroche J, Rigault P, Isabel N, Bousquet J (2012). A spruce gene map infer ancient plant genome reshuffling and subsequent slow evolution in the gymnosperm lineage leading to extant conifers. BMC Biol.

[48] Pavy N, Lamothe M, Pelgas B, Gagnon F, Birol I, Bohlmann J (2017). A high-resolution reference genetic map positioning 8.8 K genes for the conifer white spruce: structural genomics implications and correspondence with physical distance. Plant J.

[49] Rigault P, Boyle B, Lepage P, Cooke JEK, Bousquet J, MacKay JJ (2011). A white spruce gene catalogue for conifer genome analyses. Plant Physiol.

[50] Pavy N, Deschênes A, Blais S, Lavigne P, Beaulieu J, Isabel N (2013). The landscape of nucleotide polymorphism among 13,500 genes of the conifer *Picea glauca*, relationships with functions, and comparison with *Medicago truncatula*. Genome Biol Evol.

[51] Pavy N, Pelgas B, Beauseigle S, Blais S, Gagnon F, Gosselin I (2008). Enhancing genetic mapping of complex genomes through the design of highly-multiplexed SNP arrays: application to the large and unsequenced genomes of white spruce and black spruce. BMC Genomics.

[52] Prunier J, Laroche J, Beaulieu J, Bousquet J (2011). Scanning the genome for gene SNPs related to climate adaptation and estimating selection at the molecular level in boreal black spruce. Mol Ecol.

[53] Prunier J, Gerardi S, Laroche J, Beaulieu J, Bousquet J (2012). Parallel and lineage-specific molecular adaptation to climate in boreal black spruce. Mol Ecol.

[54] Pelgas B, Bousquet J, Beauseigle S, Isabel N (2005). A composite linkage map from two crosses for the species complex *Picea mariana × Picea rubens* and analysis of synteny with other Pinaceae. Theor Appl Genet.

[55] Legarra A, Misztal I (2008). Technical note: computing strategies in genome-wide selection. J Dairy Sci.

[56] Legarra A. GS3 web folder. [cited 2016 2016-07-31]; Available from: http://genoweb.toulouse.inra.fr/~alegarra/gs3_folder/.

[57] Isabel N, Beaulieu J, Bousquet J (1995). Complete congruence between gene diversity estimates derived from genotypic data at enzyme and random amplified polymorphic DNA loci in black spruce. Proc Natl Acad Sci U S A.

[58] Lenz P, Auty D, Achim A, Beaulieu J, Mackay J. Genetic improvement of white spruce mechanical wood traits — early screening by means of acoustic velocity. Forests. 2013;4:575–94.

[59] Beaulieu J, Perron M, Bousquet J (2004). Multivariate patterns of adaptive genetic variation and seed source transfer in *Picea mariana*. Can J For Res.

[60] Li P, Beaulieu J, Bousquet J (1997). Genetic structure and patterns of genetic variation among populations in eastern white spruce (*Picea glauca*). Can J For Res.

[61] Jaramillo-Correa JP, Beaulieu J, Bousquet J (2004). Variation in mitochondrial DNA reveals multiple distant glacial refugia in black spruce (*Picea mariana*), a transcontinental North American conifer. Mol Ecol.

[62] Gérardi S, Jaramillo-Correa JP, Beaulieu J, Bousquet J (2010). From glacial refugia to modern populations: new assemblages of organelle genomes generated by differential cytoplasmic gene flow in transcontinental black spruce. Mol Ecol.

[63] De Lafontaine G, Turgeon J, Payette S (2010). Phylogeography of white spruce (*Picea glauca*) in eastern North America reveals contrasting ecological trajectories. J Biogeogr.

[64] Jaramillo-Correa JP, Beaulieu J, Bousquet J (2001). Contrasting evolutionary forces driving population structure at expressed sequence tag polymorphisms, allozymes and quantitative traits in white spruce. Mol Ecol.

[65] Perron M, DeBlois J, Desponts M (2013). Use of resampling to assess optimal subgroup composition for estimating genetic parameters from progeny trials. Tree Genet. Genomes.

[66] Murray BG (1998). Nuclear DNA amounts in gymnosperms. Ann Bot.

[67] De Roos A, Hayes BJ, Spelman R, Goddard ME (2008). Linkage disequilibrium and persistence of phase in Holstein–Friesian, Jersey and Angus cattle. Genetics.

